# Improving clinical trial readiness to accelerate development of new therapeutics for Rett syndrome

**DOI:** 10.1186/s13023-022-02240-w

**Published:** 2022-03-04

**Authors:** Helen Leonard, Wendy Gold, Rodney Samaco, Mustafa Sahin, Timothy Benke, Jenny Downs

**Affiliations:** 1 Telethon Kids Institute, Centre for Child Health Research, The University of Western Australia, PO Box 855, West Perth, WA 6872 Australia; 2 University of Sydney, Sydney, NSW Australia; 3 Department of Molecular and Human Genetics, Baylor College of Medicine, Jan and Dan Duncan Neurological Research Institute at Texas Children’s Hospital, Houston, TX USA; 4 Department of Neurology, Rosamund Stone Zander Translational Neuroscience Center, Boston Children’s Hospital, Harvard Medical School, Boston, MA USA; 5 Paediatric Neurology, Children’s Hospital Colorado, University of Colorado School of Medicine, Aurora, CO USA

## Abstract

Rett syndrome is associated with severe functional impairments and many comorbidities, each in urgent need of treatments. Mutations in the *MECP2* gene were identified as causing Rett syndrome in 1999. Over the past 20 years there has been an abundance of preclinical research with some studies leading to human clinical trials. Despite this, few viable therapeutic options have emerged from this investment of effort. Reasons for this lack of success as they relate both to preclinical research and the clinical trial landscape are discussed. Considering what needs to be done to promote further success in the field, we take a positive and constructive approach and introduce the concept of clinical trial readiness and its necessary ingredients for Rett syndrome. These include: listening to the needs of families; support from advocacy groups; optimising use of existing clinic infrastructures and available natural history data; and, finally, the validation of existing outcome measures and/or the development and validation of new measures. We conclude by reiterating the need for a collaborative and coordinated approach amongst the many different stakeholder groups and the need to engage in new types of trial design which could be much more efficient, less costly and much less burdensome on families.

**Supplementary Information:**

The online version contains supplementary material available at 10.1186/s13023-022-02240-w.

## Introduction

An intriguing set of characteristics of apparently normal early development followed by a period of regression with loss of hand and communication skills and the onset of an unusual pattern of hand stereotypies first brought Rett syndrome (RTT) to the attention of the child neurology community in 1983 [1]. However it was the identification of its relationship with the *MECP2* gene that subsequently propelled RTT to the world stage in 1999 [2].

The *MECP2* gene is located on the long arm of the X-chromosome and contains 4 exons, producing two isoforms which are alternatively spliced to produce *MECP2E1*/isoform 2 (NM_001110792.1) and *MECP2E2*/isoform 1 (NM_004992.3) [3]. Isoform 2 utilises the translation start site in exon 1 and lacks exon 2, and is the predominant isoform in the central nervous system, whereas isoform 1 uses a translation start site in exon 2 and comprises of exons 2, 3, and 4. Both isoforms share the methyl binding domain (MBD), transcription repressor domain (TRD), and C-terminal domains, characteristic of the MeCP2 protein. Despite alternative splicing, both isoforms of *MECP2* are essential for normal brain development [4]. Additionally, the isoforms undergo alternative poly-adenylation, producing four different 3’ untranslated regions (UTR) lengths, which play a critical role in transcriptional regulation of *MECP2* transcripts throughout development. To date, over 800 pathogenic mutations have been detected within the *MECP2* gene [5]. These mutations include a range of missense, nonsense, frameshift, and in-frame insertions or deletions, as well as large deletions spanning whole exons or even the entire gene. MeCP2 has been implicated in a wide range of molecular functions, including transcriptional repression and activation, chromatin architecture, alternative splicing, miRNA processing and translational regulation, thus regulating a number of cellular processes, reviewed in detail recently [6, 7].

Prior to the elucidation of the genetic basis of RTT, the first epidemiological study undertaken in Texas in 1993 [8] provided a model for the subsequent Australian population-based registry study, which identified an incidence of ~ 1/10,000 female births [9]. Life expectancy has been more challenging to investigate, given the requirement for longitudinal follow-up of population-based cohorts. Using this optimal methodology, latest estimates suggest survival to nearly 60% at 37 years [10], although longer survival past the 5th decade was demonstrated in a non-population-based US study with shorter follow-up [11].

Since its first identification, it has been clear that there is considerable variability within the RTT phenotype [12]. However, it was more than a decade later when the detailed early clinical descriptions [12] (e.g. formes frustes, late regression, congenital variants, early onset seizure) were able to be matched to specific *MECP2* or other (*FOXG1* & *CDKL5*) genotypes [13]. To reach this point required the undertaking of population-based[14] and large sample sized studies[15, 16] across the globe.

Building on the work of Katz and co-authors in 2016 [17] and the comprehensive cataloguing by Gomathi et al. [18] of almost a hundred pre-clinical and clinical studies already undertaken in RTT, our review first summarises the comorbidities affecting individuals with RTT and what we know about their management. We then briefly describe the current landscape of clinical trials starting with preclinical research and moving on to human trials focussing on those published or being conducted since 2016, but also considering the status of gene therapy. An important purpose of this review is to identify what is needed in terms of clinical trial readiness in order to accelerate the development of successful therapeutics for RTT. The roles of database infrastructure, natural history data, coordinated trial networks and patient advocacy groups are discussed followed by specific discussion on consumer expressed needs. The other key focus is on outcome measures and their need for validation, both biomarkers and parent-reported outcome measures including those that are RTT-specific and those that are generic. The review concludes with a section on the value of a collaborative model in ensuring successful translation of therapeutics into the clinic.

## Comorbidities and their management

Although clearly a genetically based neurological disorder causing severe functional impairment, RTT is also associated with multiple comorbidities which prompt clinical presentation and need for treatments [19]. These comorbidities affect multiple systems (neurological, gastro-intestinal, cardiac, endocrine and orthopaedic)[20] appear at varying ages but may not be present in early childhood when regression of skills and stagnation in development are of primary concern to caregivers. One exception is gastro-intestinal issues specifically feeding difficulties, constipation and reflux which may present early and for which clinical management guidelines are available [21]. Growth is also impaired from an early age but with weight and height both affected nutritional status may not always be an obvious early concern [22]. However, enteral feeding through gastrostomy is becoming increasingly available to young children with RTT and families appear to generally welcome this option [23]. Although gastrostomy improves nutritional status there is no evidence to date of any associated improvement in life expectancy [24].

RTT has been termed as a Developmental Encephalopathy (DE) and a comparison with three other DEs, *CDKL5* Deficiency Disorder (CDD), *FOXG1* disorder & *MECP2* duplication syndrome (MDS) found that severity was greatest in CDD [25]. Age of seizure onset at 4–5 years in RTT is considerably later [26] than in CDD, although earlier than in MDS [27]. Reports on the prevalence of epilepsy in RTT are variable and range from as low as 48% when clinician diagnosed [28] to as high as 81% and 90% in Australian and US studies [26, 29]. The rate of active epilepsy appears highest in the 12–17 year age group but varies by genotype [28, 30, 31]. Its impact may also be variable with a third of individuals being seizure free and about a third having drug-resistant epilepsy [31]. To date there have been no studies comparing the effects of different antiseizure medications (ASMs) in RTT. A randomized controlled trial (RCT) to investigate the efficacy and safety of oral cannabidiol (NCT03848832) did not evaluate seizure frequency as one of the outcomes.

Autonomic disturbances presenting as awake breath-holding and/or hyperventilation are a characteristic and troublesome feature of RTT, but research in animal models appears to exceed what is known in humans [32]. Even the prevalence of this comorbidity is unclear. In a study using the InterRett database, time-to-event analysis showed almost two thirds would have experienced breath-holding and a half hyperventilation by the age of 5 years with ongoing impact greatest for those with a Arg294* mutation [33]. Findings from a US study were consistent [34]. In the absence of any available treatment for this challenging co-morbidity, a clinical trial of sarizotan (NCT02790034) prompted by promising results from animal studies[32] was undertaken. Unfortunately, the primary endpoint, a percentage reduction in episodes of apnea during waking time, was apparently not met for the intervention group compared with placebo [35].

While autonomic disturbances are relatively unique to RTT, sleep disturbances, although also common in children with other neurodevelopmental disorders [36], have always been included amongst the supportive criteria [37–39], including in the most recent revisions in 2010 [39]. Successive studies have highlighted difficulties initiating and maintaining sleep [40–42], while in subsequent research adverse associations between child sleep disorders and parental wellbeing have also been found [43, 44]. As with autonomic dysfunction there are associations with genotype and individuals with a large deletion or p.Arg294* have been shown to have more severe sleep disturbances [41, 45]. There are no published trials investigating treatments for sleep disturbances in RTT, and observational data could not demonstrate any further benefit from use of melatonin than could be achieved from the judicious use of sleep hygiene practices [42].

Musculoskeletal issues have a major impact on the health and likely quality of life of girls with RTT. Scoliosis is one of the few comorbidities where there is an intervention, albeit surgical, which indeed brings benefits beyond the primary aim of treatment [46]. It is also one of the few areas where there has been an attempt to develop specific guidelines to improve clinical management [47]. Another is bone health where there are considerable opportunities for prevention [48].

Informed by a literature review and developed using a Delphi process as were the previous guidelines [21, 47, 48], a recent publication has catalogued comprehensive lists of recommendations for management of the comorbidities associated with each body system [19]. These recommendations should be helpful for clinicians in the field, whilst we await the development of a better evidence base for future treatments for specific comorbidities in RTT.

## The current landscape of clinical trials in RTT

### Pre-clinical research and its role in informing human clinical trials

**Fig. 1:**
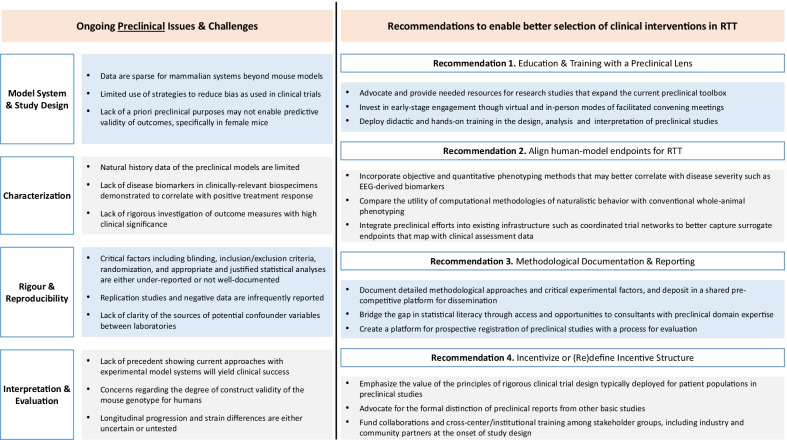
Limitations of and possible solutions for the use of experimental model systems for pre-clinical studies

Multiple pre-clinical experimental systems for RTT have been developed. These include cellular models and animal models including mice (knockout, knockin and mutation specific), rats and the cynomolgus monkey [49, 50]. For decades mouse models have represented the gold standard in therapeutic discovery and the existing mouse models robustly phenocopy many features of RTT including cognitive dysfunction, ataxia, breathing abnormalities, seizures, small brain volume and reduced lifespan [51]. Recently, studies using patient derived novel stem cell-based disease models such as induced pluripotent stem cells (iPSCs) have improved our understanding of disease-causing mechanisms, including disease progression, cellular changes, and altered signalling pathways in RTT, providing a more human centred approach to disease understanding and therapy assessment [52]. However, despite the promising effects identified in these iPSC models, they have not yet translated to improvements in symptom status in humans that are either consistent or substantial [52]. Critical evaluation of how preclinical studies are conceptualised, conducted and analysed informs how we can improve applicability to human trials in the future. (Fig. 1).

The most effective preclinical research practices in the RTT field have yet to be realized, lending strength to the argument that animal models, specifically rodents, may be useful but limited tools when studied in isolation. Identifying actionable translatable interventions for either core features of RTT or for specific *MECP2* mutation subtypes will depend on the experimental strategy. Indeed, recent findings from the sarizotan (NCT02790034)[35] study suggest the need for new or different approaches that do not rely on our conventional framework for advancing potential therapeutics for RTT. As one example, the decision to further develop an intervention based on animal model studies may benefit from deploying an independent and equally rigorous community-based advisory review process of the preclinical findings. In the context of sarizotan and other drugs that did not succeed in clinical trials, it is conceivable that a process as described would have led to recommendations to conduct additional follow-up replication studies with *Mecp2* rodent models across laboratories, incorporating increased sample sizes and treatment arms or duration. In principle, this level of evaluation would serve as a means of acknowledging the strengths and weaknesses of the model-based data in the therapeutic development process, however, the incentives to execute a framework in this manner are highly diverse and complex. Moreover, although the investment of time and resources necessary to fully develop a preclinical review infrastructure can be streamlined, the process and subsequent recommendations of possible follow-up work would not necessarily guarantee success in clinical trials as we explore in greater detail in the following sections.

### Human clinical trials

Thus far, clinical trials testing therapeutics in RTT have mainly focussed on symptom relief. These approaches have been divided into three categories of pharmaceuticals: drugs that target neurotransmitter systems in the brain, drugs that promote brain growth and development, and drugs that target other affected systems e.g. energy metabolism and protein synthesis.

These trials have mostly produced null, limited or modest effects. Desipiramine, a noradrenaline uptake inhibitor, appeared promising in preclinical studies[53] as an agent to reduce breathing abnormalities but a phase 2 clinical trial (NCT00990691) with 34 patients reported a null effect [54]. Sarizotan, a 5-HT1a agonist and a dopamine D2–like agonist/partial agonist associated with reduced apnoeas in the mouse model of RTT [32], has also shown a null effect on all primary and secondary outcomes in a recently completed phase 3 RCT (NCT02790034) [35]. A phase 2 trial evaluating dextromethorphan (NCT00593957) found little effect on overall clinical severity but some modest improvements in seizure spike counts for the subset who were not seizure free. Receptive but not expressive language was improved on the 5 mg but not the 2.5 mg dose, and behavioural hyperactivity measured with the Aberrant Behavior Checklist was also reduced for those on the lower dose [55].

One of the effects of impaired MeCP2 function is reduced production of Brain Derived Neurotrophic Factor (BDNF), a protein required for normal neuronal development and brain function and implicated in dendritic arborisation and synapse transmission with some clinical evidence of a role in RTT pathogenesis [56]. Aiming to reduce the deregulation of BDNF and based on effects of administration to male *Mecp2*^−/y^ mice[57], fingolimod (NCT02061137) has been shown to be safe in the six girls with RTT studied [58]. A preliminary open label trial evaluating mecasermin (rhIGF-1) (NCT0125331) found reduced apneas and improved mental health [59], but these symptoms worsened in a later placebo-controlled clinical trial (NCT01777542) [60]. Although none of the eight primary outcome measures in this phase 2 trial showed improvement, there was clinically meaningful improvement in a secondary outcome measure of social communication and improvement (lessening) of hand stereotypies, also a secondary outcome measure, but this was not considered clinically meaningful [60]. The tripeptide form of IGF-1 known as trofinetide was first evaluated in a phase 2 adult trial (NCT01703533)[61] and subsequently in a phase 2 paediatric trial (NCT02715115) confirming safety and tolerability and demonstrating improvements [62] now leading to the current phase 3 confirmatory Lavender trial (NCT04181723). While trials of ketamine (NCT03633058) and triheptanoin (NCT02696044) are still recruiting, the only other currently active phase 3 study is a double-blind, randomized, placebo-controlled, safety, tolerability and efficacy study of the Sigma-1 receptor agonist, blarcamesine (NCT04304482). This was leveraged off preclinical research ameliorating neurologic impairments in a RTT mouse model [63].

## Future prospects: gene therapy

Precision therapeutics such as gene therapy have greater potential to change disease status rather than modify symptoms, as they are targeting the root cause of the disorder. Recent success in this field is exemplified by the spinal muscular atrophy trial of ‘Zolgenzma’ [64]. Effective strategies for gene therapy include gene replacement, gene editing, RNA editing and inactive X-chromosome reactivation and are based on either viral or non-viral gene delivery to target cells [65]. Among the viral-based vector systems, adeno-associated virus (AAV) vectors have demonstrated the greatest clinical success for in vivo gene delivery, with retroviruses, lentiviruses, adenoviruses, and herpes simplex viruses also being used as successful vectors in clinical trials. The leading vector, adeno-associated virus, has been linked to dorsal root ganglion pathology in non-human primates, but this could potentially be mitigated [66, 67]. Non-viral vectors such as lipid-based, polymer-based, and inorganic-based nanoparticles are proving to be alternative delivery vehicles which may alleviate viral-based insertional mutagenesis, multi-organ toxicities, and immunogenicity concerns of the medical research community. Supplying a working copy of the coding region (cDNA) of *MECP2* to brain cells of patients to produce a functional protein would appear an obvious gene therapy approach. However, excessive expression of exogenous MeCP2 can also exacerbate disease, as observed in *MECP2* duplication syndrome and in transgenic mice overexpressing MeCP2 [68, 69]. Recent *Mecp2* gene transfer studies have confirmed these concerns, where, despite showing extended lifespan, the studies identified dose-dependent toxicity and variability in safety and efficacy issues [70]. This is highly relevant to female patients, all of whom have mosaic expression of *MECP2* (due to X chromosome inactivation)*,* as introducing another copy of *MECP2* into cells already expressing the wild type allele of *MECP2* may cause a detrimental overdose effect. Encouragingly, several pharmaceutical companies have committed to gene therapy programs for RTT with plans to initiate clinical trials possibly as early as 2022.

## Ingredients of clinical trial readiness in RTT

**Fig. 2:**
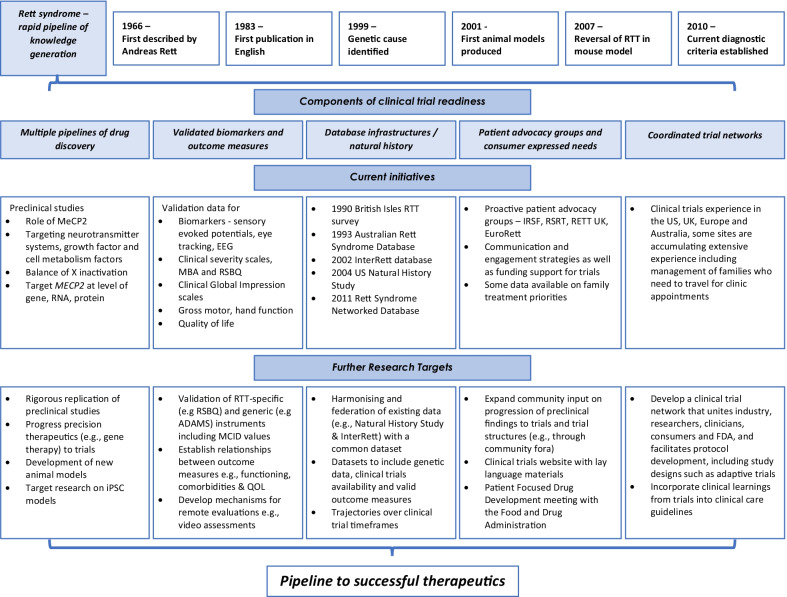
A knowledge to translation pathway for achieving successful therapeutics for Rett syndrome

*Clinical trial readiness* is a concept beyond simply having new and promising therapeutics. Beyond new therapeutics, other contributing factors include knowledge of natural history, and the distribution of genotype in populations of affected individuals, the presence of coordinated trial and care networks, knowledge of what outcomes are important to address for caregivers and families, and validated outcome measures capable of identifying meaningful change. Meaningful change, often termed minimal important difference or minimal clinically important difference (MCID), is a pre-defined key benchmark of change determined to be important to individuals that can be objectively measured [71]. The rare disease arena has shown leadership in this regard. For example, the TREAT-NMD, a global alliance formed in 2007, has been active in the establishment of large international registries, focusing initially on Duchenne muscular dystrophy and spinal muscular atrophy[72] but also including substantial achievements for less common disorders such as myotonic dystrophy[73] and autosomal recessive limb girdle muscular dystrophies [74]. TREAT-NMD registries have many roles including trial planning, recruitment and collection of natural history data, including capacity to link individuals with specific genetic variants to trials evaluating emerging therapies for such genotypes [72]. In the field of neurodevelopmental disorders, the Fragile X community has shown leadership in response to early trials failing to meet their primary endpoints, despite promising pre-clinical data and success in early-phase studies. The National Fragile X Foundation has developed a Clinical Trials Committee structure made up of a Fragile X consortium of researchers and clinicians, expert Fragile X trialists, outcome measure experts in the field, and family stakeholders to assist and support new treatment developments [75]. This consortium is designed to be highly collaborative and is mindful of previous experiences of patients in unsuccessful trials and the limited resources available to a rare disease community. The Fragile X community advises industry to engage with their trial community collaboratively with the aim of optimising trial success [75]. Figure 2 demonstrates the ingredients of clinical trial readiness and has been developed in response to the learnings from these other disorders as well as experience in RTT.

### Database infrastructure

The pipeline of discovery in RTT has included establishment of registry infrastructures seeking to understand epidemiology, natural history and life expectancy [13]. These include the Australian Rett Syndrome Database [9], which is population-based and longitudinal; the international InterRett database, a large dataset enabling genotype phenotype studies[14]; the US Rare Disease Consortium Research Network for RTT collecting clinical and parent reported data across US sites (NHS, U54 HD061222; NCT00299312/NCT02738281[15]); and the European Rett Networked Database [13, 76]. These infrastructures have accumulated vast datasets and illustrate global efforts that have sought to understand the trajectories of symptoms, genotype phenotype relationships, and insights into the effectiveness of some clinical treatments. These achievements have required extensive infrastructure, the ingredient of time, and are not easily replicated. The TREAT-NMD database system contains harmonised data that is federated, such that national registries exist but contribute to a central hub whilst retaining ownership of their data [72]. Rett syndrome could profit from such a federated system which has also provided considerable benefit in autism research [77].

### Natural history data

Disease trajectories in RTT are becoming more evident because of these infrastructures. Data from 1052 participants collected during 4940 clinical visits to the US Natural History Study (NHS) showed increased clinical severity with time regardless of clinical severity at baseline, but with variation by genotype [15]. These durations of observations are likely to be over a longer time frame than most clinical trials. The Australian Rett Syndrome Database, maintained now for 27 years with seven waves of follow-up data collection, provides additional opportunities for examination of longitudinal change. Scoliosis is the most common orthopaedic comorbidity, observed in 4286 person-years of Australian data to have a median age of onset of 11 years and an increase in Cobb angle of approximately 5 degrees per year [78]. Sleep disturbances such as night waking, night screaming and night laughing are prominent and trajectories observed from 2000 to 2011 indicate consistently high prevalence throughout childhood with a small decline into adulthood [41]. Using the same data source, it was possible to show that growth parameters improved over time and that BMI was greater in those with gastrostomy insertion[24] as well as to investigate the complex interplay with parental wellbeing [43].

These trajectories’ data are important and highlight the persistence or worsening of problems with increasing age. However, no data are available on trajectories that would be expected over the duration of a clinical trial, although some test retest reliability data collected over shorter periods suggest stability. For example, gross motor skills measured using the Rett Syndrome Gross Motor Scale were consistent over 1-week [79]. The Quality of Life Inventory-Disability (QI-Disability) was developed and validated for children with intellectual disability including RTT, and scores measured after one month were similar, taking into account changes in physical health and behaviour [80]. No other literature indicates the stability or change in motor skills or comorbidity status over time periods such as six months, as would be observed during a clinical trial. It is critical that these data are collected, to inform planning of clinical trials and potentially, for use as historical control data to enable more rapid accrual of an adequate sample size. Careful choosing of outcome measures is also critical. The Fragile X experience has also suggested value in including multiple endpoints in trial protocols combining biomarkers with parent reported measures, and selecting aggegate or domain-specific measures based on factors such as the hypothesised mechanism of effect or the heterogeneity of the population [81].

### Coordinated trial networks are under development

The majority of trials have been conducted in the US, drawing particularly on the resources of the clinics contributing to the 16-year NHS. All clinical trials experience is preparatory for the clinical trials of the future, relevant to ensuring that infrastructure and staff have optimal expertise in protocol administration and compliance, including supporting families, many of whom will travel to attend the participating clinic on account of the rarity of the disorder. The recent sarizotan trial (NCT02790034) included sites in the US, Australia, India, Italy and the UK. Going forward, this multi-national approach is important to foster for a rare disease, to expand the pool of eligible participants and ensure that families across more geographical locations have access to clinical trials. It is critical for the community to learn lessons from previous clinical trials, including how they were conducted, and to identify the successful elements as well as the limitations [81]. For example, some lessons learned from unsuccessful trials for Fragile X have suggested that further attention be paid to measurement and potentially longer trial duration to capture longer term cognitive and adaptive changes [75]. A means of facilitating such communications could be the establishment of a consortium or hub which would allow interchange of ideas and resources as well as data sharing amongst sectors and the building of relationships with the Food and Drug Administration (FDA) or European Medicines Agency (EMA)(see Fig. 2) [81, 82].

### Patient advocacy group (PAG) support

RTT Clinical trials are actively supported by advocacy organisations including the International Rett Syndrome Foundation, the Rett Syndrome Research Trust, Rett UK, AiRett and the Rett Syndrome Association of Australia among others. Family voice has had a foundational role in enabling clinical trials for RTT and their ongoing fundraising support has ensured a continuing pipeline for the development of new therapeutics and new clinical trials.

### The voices of caregivers: consumer expressed needs

Despite the severe disability and clearly reduced life expectancy in RTT there is minimal available research on the issues of most concern to families and their treatment priorities. This information is integral to decisions by biopharmaceutical companies as to what should be the targets of clinical trials. When using InterRett data to investigate autonomic dysfunction, the impact of abdominal bloating surpassed even that of breath-holding and hyperventilation with almost half the parents reporting a moderate or major impact for those experiencing this condition [33]. Just over a third of caregivers reported that sleep problems had a moderate or major impact on their child and a slightly higher percentage reported an impact on themselves [42]. Moreover, in an InterRett study recently undertaken [83], caregivers were specifically asked to describe their three major concerns for their children’s physical and emotional health (unpublished data). The concerns expressed were then grouped into one of nineteen domains representing function, comorbidities, mental health (e.g.anxiety, depression, challenging behaviours), participation and quality of care. The greatest concern by far related to the child’s communication ability, for which management guidelines have recently been published [84], followed by lack of participation and ability to walk. Symptoms of depression and uncontrolled seizures were also major concerns. These concerns need to be formally evaluated to provide specific guidance.

Engagement strategies that involve both consumers and stakeholders at all stages from drug discovery to clinical implementation are necessary to progress the understanding of consumer needs and views. As only preliminary information is available thus far, further granularity is crucial in understanding the main concerns that families believe should be targeted by new therapeutics. An additional mechanism of achieving caregivers’ perspectives, at least in the USA, is through a Patient-Focused Drug Development (PFDD) meeting (see Fig. 2) as has been done recently by the CDKL5 Deficiency Disorder community [85]. PFDD meetings are designed by the US Food and Drug Administration (FDA) to allow patients and, in the case of RTT, caregivers to elicit their perspectives on the most significant symptoms of the condition and its impact on daily life as well as current treatments being received. Such meetings often spearheaded by Patient Advocacy Groups can be attended by patients, caregivers, clinicians, researchers, industry and FDA representatives.

Parental concerns may vary with the age, genotype or level of functional ability of their child. The amounts of change that are important to achieve with any new therapeutic, otherwise known as the MCID [71], have not yet been identified by domain in RTT, and consumer involvement in these processes is critical. Validated outcome measures for relevant domains are essential for clinical trial readiness and best practice methodologies for their development and validation include involvement with consumers [86]. For planning clinical trials, consultation with consumers and stakeholders in the planning of recruitment and evaluation of the study design is important at an early stage. Consumer perspectives can help to ensure that trial processes are feasible, acceptable and are not overly burdensome. Coordination between patient advocacy groups, consumers, clinicians, researchers, industry representatives and regulatory bodies (e.g. the FDA’s Center for Drug Evaluation and Research) is a critical component in the pipeline to successful therapeutics (see Fig. 2). This could be achieved by the establishment of a consortium or hub where ideas and resources for both pre-clinical (see Fig. 1) and clinical studies including outcome measures that have been developed or validated can be shared and mechanisms developed for data harmonisation and federation [82].

## Validated outcome measures to assess efficacy of new therapeutics

**Fig. 3:**
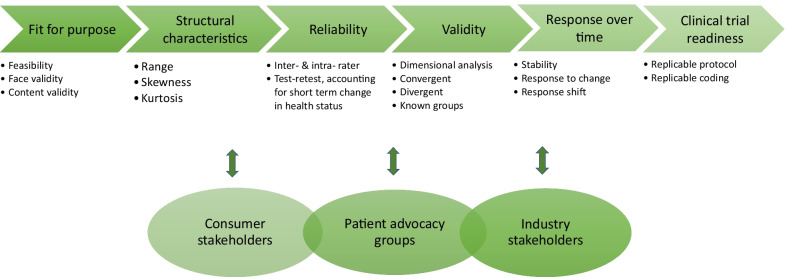
Pipeline to clinical trial readiness

Capacity to collect valid information on objective and subjectively reported outcomes is essential to enable clear monitoring of clinical progress and rigorous evaluation of new therapeutics. Both biomarkers and parent reported outcome measures (indicating how the child feels and functions) contribute to knowledge of natural history and quantification of any treatment effect. To be considered valid, an outcome measure needs to be appropriate for purpose, feasible to administer, and supported by satisfactory validity, reliability and responsiveness to change data, consistent with FDA guidance (Fig. 3) [87]. Limited use of validated outcome measure in RTT clinical trials to date has posed substantial threat to trial validity (see Additional file 1: Table S1 for documentation of the use of clinician and parent reported outcome measures in clinical trials and Additional file 2 for references pertaining to Table S1).

### Biomarkers

Several recent clinical trials, not just in RTT but also in other neurodevelopmental disorders, [81] have failed to show efficacy despite very promising findings in animal models. These failures are potentially related to our inability to adequately stratify patient populations, for example by genotype where we know that mutations such as p.Arg255* and p.Arg270* are severe while C-terminal deletions, p.Arg133Cys, p.Arg306Cys and p.Arg294* are generally milder[14] or measure target engagement in the tissues of interest such as the brain. Thus, there is an urgent need to develop, test and validate useful clinical biomarkers in RTT that bridge human and animal studies. There are a number of potential biomarkers currently being studied including electrophysiological [e.g., EEG/MEG measured event related potentials (ERPs) and oscillations], imaging (structural and functional MRI), functional [e.g., continuous monitoring with wearable sensors, eye tracking, pupillometry, transcranial magnetic stimulation (TMS)] and biochemical/molecular ‘fluid’ biomarkers.

There are accumulating data indicating that individuals with RTT and animal models display atypical responses on electrophysiological assessments of brain function. Sensory evoked potentials and EEG analysis have been utilized in several preclinical and clinical studies in RTT (for an exhaustive review, see [88]). Work by Foxe et al. 2016 [89] demonstrated that timing and morphology of auditory evoked potentials are abnormal in girls with RTT compared to typical developing controls. A mouse model of RTT also shows a similar difference [90]. Artoni, Fagiolini and colleagues discovered that MeCP2-deficient mice have strikingly abnormal late phase visual evoked potentials suggesting abnormal cortical processing [91]. Visual evoked responses obtained in girls with RTT demonstrated the same defect and led to a new understanding of visual defects in the girls that had not previously been recognized [92]. Furthermore, the degree of abnormality of evoked responses correlates with overall severity of the disorder. Loss of *Mecp2* in peripheral mechanosensory neurons is associated with tactile and social interaction deficits in mice [93], but it is not yet known whether similar phenomena occur in individuals with RTT. More recently, Artoni also reported that *Mecp2* heterozygote awake adult female mice exhibit abnormal spontaneous pupil fluctuations, indicating impaired cholinergic neuromodulation and altered arousal state [91]. By training a neural network on mouse pupillometry data and using human heart rate variability as a surrogate for arousal, the investigators were able to detect the same arousal alteration in RTT patients compared to typical developing controls. These findings indicate that both sensory and autonomic nervous system physiology could be cultivated for developing biomarkers for RTT intervention trials. Moreover, ERPs have been confirmed to correlate with severity and demonstrated to be stable with time [94] typically associated with a clinical trial. However, improvement of any biomarker and correlation with symptomatic improvement in pre-clinical animal treatment models has not yet been demonstrated.

Biomarkers can be used as an objective proxy for (a) predicting response prior to treatment (biomarker suggests patient will respond) and/or (b) anticipating future response to treatment (biomarker predicts that treatment is working before change seen in measure) and/or c) biomarker change correlates with measure change after treatment. To date only a few RTT intervention trials have employed biomarkers and tried to correlate them with clinical improvements, as in the third scenario, with mixed results. The placebo-controlled clinical trial of mecasermin (rhIGF-1) (NCT01777542) used EEG-based biomarkers and a number of parent-completed scales [60]. In this study worsening of symptoms in the parent-completed scales paralleled deterioration in EEG parameters. The dextromethorphan (NCT00593957) study, as described earlier in the text, showed improvements in seizure spike counts, but these changes were not reflective of a decrease in clinical severity [55]. Two currently active intervention trials are utilizing EEG as a biomarker: ketamine (NCT03633058) and triheptanoin (NCT02696044). The correlation between the biomarker and clinical assessments will require an iterative process through clinical trials. For example, beta oscillations in Dup15q syndrome appear to show correlation with clinical traits such as epilepsy and Vineland Adaptive Behavior Scale scores [95, 96]; however, it is not clear whether these oscillations will change with intervention trials that additionally improve epilepsy and behavioral outcomes. Delta power in EEG recordings has also very recently been shown to provide a robust biomarker for cognitive function in Angelman syndrome [97].

In summary, several studies to date provide objective evidence for deficits in sensory processing across multiple modalities in RTT and provide the scientific premise for pursuing whether these noninvasive and objective biomarkers can be informative in future clinical trials. However, as with other neurodevelopmental disorders, further research is still needed outside the confines of a clinical trial to confirm relationships between biomarkers and clinical outcome measures including those that are parent-reported.

### Outcome measures developed for RTT

Clinical severity**:** The clinical severity scores currently in use[13] were originally developed for the purpose of evaluating genotype–phenotype relationships [14, 16]. However, their reliability, factor structures and responsiveness to change within a clinical trial timeframe have not been examined. In the phase 2 adult trial evaluating trofinetide [61], a subset of five items from Rett Syndrome Clinical Severity Scale (CSS) were selected to form a “Change Index” with no change observed following trofinetide use. The Kerr Severity Scale was used in the phase 2 rhIGF-1 (mecasermin) trial (NCT01777542), with some worsening of scores [60]. Validation data for the Motor-Behavioral Assessment (MBA), which includes some historical aspects of regression, has only just become available [98]. Seventeen items that could be amenable to change were selected from the MBA to create the MBA “Change Index,” and non-significant improvement was demonstrated in the phase 2 paediatric trofinetide trial (NCT02715115) [62]. Additional studies are required to confirm sensitivity of the MBA to change.

The Clinical Global Impression scales describe severity or change of severity for specific symptoms and have recently been adapted to RTT [99]. This process involved defining seven category descriptors across each rating scale for the domains of communication, ambulation, hand use, use of eye contact, autonomic function, seizures, and attentiveness [99]. However their use requires familiarity with RTT that limits use to major clinical centers and may be therefore be difficult to translate into wider use. As the authors recognised [99], more complete validation studies are still awaited. Improvement in the CGI-I was found in the paediatric trofinetide trial (NCT02715115) [62].

Socio-emotional domain: The Rett Syndrome Behaviour Questionnaire (RSBQ) was developed as a diagnostic tool to clinically differentiate females with RTT from those with other severe intellectual disability [100]. Individual domains have been used successfully in a genotype–phenotype study to assess mood and anxiety [101], Although it was not designed to measure change in a clinical trial and has not been validated for this purpose, in the absence of any other Rett-specific instruments the RSBQ has been used as an outcome measure in clinical trials. Scores for the fear and anxiety subscales slightly worsened in the rhIGF-1 trial (NCT01777542) [60], while improvement in the total RSBQ was found in the paediatric trofinetide trial (NCT02715115) [62]. Subsequent to this, the measurement properties of the RSBQ were reported to be poor in a study [102] which was itself criticized for inadequate representation of the population, a clinically heterogeneous sample and missing psychometric evaluations [103]. In an earlier study test–retest and intra-rater reliability for the anxiety-related domain were found to be adequate but poorer than for the other instruments, the Anxiety Depression and Mood scale and the Aberrant Behaviour Checklist-Community [104]. Thus it would seem that the RSBQ still has potential as an outcome measure but needs further analyses to characterise its profile and identify options for improvement [103].

Functional ability domains: Some measures for specific domains are also available. The 15-item Rett Syndrome Gross Motor Scale (RSGMS) although not frequently used, has undergone substantial validation including evaluation of factor structure and test–retest reliability [105]. It has been used in a single case design study [106] and a randomized stepped wedge design study [79] investigating therapy interventions where both studies achieved a change in total score greater than the minimal detectable difference. The Rett Syndrome Clinician Rating of Ambulation and Gross Motor Skills (RTTAMB) and the Rett Syndrome Clinician Rating of Hand Function (RTT-HF) are being used as secondary outcome measures in the Lavender study (NCT04181723) but for neither is there any published validation data. However, a measure, comprising eight categories of hand function and thus providing more granularity than the clinical severity scales, has also been developed and is available [107]. Whether new pharmaceutical therapeutics for RTT can achieve changes in gross motor or hand function is not known, but this certainly would be a goal of gene therapy where the high quality and validity of the outcome measures will be paramount to the success of any trial.

### Generic outcome measures

The Mullen Scales of Early Learning (MSEL) is a generic measure of developmental skills and was a secondary outcome measure for the phase 2 rhIGF-1 trial (NCT01777542) [60]. Modest improvements in the Receptive Language domain of the MSEL were initially reported for the Dextromethorphan trial but not replicated in a follow up study (NCT00593957) [55]. Whilst not yet validated for RTT, its administration and scoring have now been adapted to account for the presence of dyspraxia and capacity to use eye gaze and right skew was less apparent for the visual reception and receptive language domains [108]. This version is yet to be included in a clinical trial protocol and further validation and examination of responsiveness to change are needed.

The Communication and Symbolic Behavior Scale-Developmental Profile (CSBS-DP) measures early communication and symbolic skills in children under two years [109]. It was used as a secondary outcome measure for the rhIGF-1 trial (NCT01777542), with improvements observed in the social domain [60]. The Aberrant Behaviour Checklist (ABC) and the Anxiety, Depression, and Mood Scales Anxiety, Depression and Mood Screen (ADAMS)[110] measure socio-emotional behaviours [111]. Finally the Vineland Adaptive Behavior Scales (VABS) measures adaptive functioning although concern has been raised about the floor effect where a large proportion of individuals in more severely affected populations score poorly [112]. Whilst each measure has some validation data for intellectual disability, psychometric investigations for RTT are needed if they are to continue to be used.

Quality of life (QOL) is a concept that articulates how well an individual is living, reflecting closely patient feeling and functioning, and hence it is a critical outcome for evaluating interventions. Generic QOL measures have been used in RTT clinical trials but may not be appropriate because their development was not based on the domains of QOL important for children with intellectual disability [113]. There are however some validation data for the Child Health Questionnaire-P50 in RTT [114]. As expected, physical summary scores were poorer for individuals with genotypes associated with greater clinical severity, while psychosocial summary scores were poorer for individuals with less severity but more behavioural challenges. Based on extensive qualitative data, the Quality of Life Inventory-Disability (QI-Disability) was developed specifically for children with intellectual disability and has stood tests of reliability, validity and responsiveness to change [80, 113, 115]. Thus far in RTT, QI-Disability has been used in a pilot study evaluating activity programs for RTT (NCT03848442) and is currently an outcome measure in a follow up clinical trial (NCT04167059).

It is clear that a core set of outcome measures is lacking for RTT and that development and validation studies are lagging behind preclinical and clinical research. For most outcome measures in frequent use, poor validation data are available. Thus far also, few comorbidities beyond epilepsy have been evaluated [55] and the choice of BioRADIO wearable technologies to measure objectively the patterns and regularity of respiratory function (NCT02790034) was based on small observational studies [116]. To our knowledge, however, there have been no published studies investigating any relationship between these autonomic measures and parent-report data, when assessed contemporaneously. Accordingly, there are many research imperatives in this arena. Further data are needed to combat the strong historical influences on use of unvalidated outcome measures, the pressing need for systematic evaluations of the validity, reliability, responsiveness and the MCIDs of available and new outcome measures. We would expect that measures of functional ability such as those for hand and gross motor function would be particularly suitable for disease-modifying genetic trials while assessment of comorbidity status (e.g. seizure diaries) will indicate effects in drug studies. Measuring QOL is essential for all types of studies because it can capture change in the day-to-day living of a child. When validation data are satisfactory, it will be imperative to collect natural history data over clinical trial timeframes of approximately a six-month period, so that trial findings can be compared to natural history.

## A collaborative model for future success

Many of the ingredients for clinical trial readiness are already in place: there are active programs of pre-clinical research, a powerful history of database development which has enabled the availability of some trajectory data, and there is growing clinical trials experience, particularly in the US. Considerable work is, however, still necessary to achieve a core set of validated outcome measures for RTT.

It is important to note that RTT is not alone in the challenges faced in translating advances in understanding the biology of neurodevelopmental disorders into effective treatments in the clinic. For example, the need for rigorous execution and transparent reporting for data from preclinical models, dynamic and clinically meaningful outcome measures and use of biomarkers to demonstrate target engagement apply not only to RTT but also to many genetic disorders in which novel therapeutics are being developed. In an ideal world, development of outcome measures, especially MCIDs, must be established prior to therapeutic trials. However, the current situation where lack of the ideal does not prevent otherwise good trials from proceeding will continue. The risk is more likely that modestly helpful but important therapeutic interventions will be interpreted as failures. The lessons learned from RTT apply to a number of genetic conditions associated with autism and intellectual disability, such as Fragile X Syndrome and Tuberous Sclerosis Complex. Furthermore, given the rapid growth in diagnostic tools for genetic disorders, the foundational knowledge gained would be instructive to the process of N-of-1 studies which continue to attract considerable attention in the rare disease space [117]. Conversely, findings from other disorders will help shape better clinical research in RTT [75, 81, 118–120]. Our comments about biomarkers, outcome measures, clinical trial design will likely have similar impact across the field of neurodevelopmental disorders.

For the future, it is imperative that the RTT community, including industry, consider alternative ways of working together for agile evaluation of new therapeutics. For example, there are increasing demands on available funding, the SARS-CoV-2 pandemic requires different models of conducting business and the recent Newron STARS trial (NCT02790034) illustrated capacity to work across multiple continents to achieve the required sample size. Supported primarily by the US National Cancer Group (thecogfoundation.org), the Children’s Oncology Group is a collaborative international group dedicated to overseeing clinical trials and contributing to improving clinical care and outcomes for children’s cancer. The Clinical Trials Committee established by the National Fragile X Foundation could also be an important model for Rett syndrome [75]. Could similar structures be possible for RTT and what linkage with other neurodevelopmental disorders could be fruitful? United within an academic and patient advocacy structure, informed by consumer experience and partnering with industry, a neurodevelopmental trials consortium could oversee and coordinate a broad clinical trials program to evaluate known and new therapeutics, potentially for more than one disorder, providing training for a range of trial sites and supporting use of new trials design such as Adaptive Platform Trials (APTs).

Researchers, clinicians and the RTT community acknowledge that traditional, free-standing, parallel-group RCTs are time-consuming, burdensome and costly, and it is challenging to achieve adequate sample size for the evaluation of subgroups [121]. APTs offer an alternative structure that is agile and responsive, where multiple interventions are evaluated within the one trial, randomisation schedules and treatment doses can be adaptive, and separate effect estimates can be generated across subgroups of participants [121]. Each therapeutic enters and leaves the platform on the basis of a predefined decision algorithm which reflects thoughtful clinical planning and decision making. It is imperative that MCIDs for outcome measures be identified for RTT to enable clear decision making of whether to remain with the therapeutic or to change to an alternative therapeutic. The APT approach minimises downtime between trials and could evaluate both new and currently used therapeutics with a poor evidence base.

Finally, rare disease communities are hungry for translational scientific evidence to provide relief to the burden imposed by RTT. When transformative therapeutics are identified, how will manufacturing capacity be ensured to meet demand and how will the cost be managed to enable broad accessibility across groups within and between countries, beyond the principle that goods should only be available to those who can pay? Progress along the pipeline of discovery has been strong since RTT was first described in 1966. In this current era of neuroscience and clinical trials, there is an imperative to learn from errors of the past, build a well-validated set of outcome measures and biomarkers, and consider novel ways of collaborating to challenge the current boundaries for current clinical management of RTT.

## Supplementary Information

**Additional file 1: Table S1.** The use of outcome measures in clinical trials in Rett syndrome.

**Additional file 2:** References for Table S1.

## Data Availability

Not applicable.
