# Supplementary material for: Improving clinical trial readiness to accelerate development of new therapeutics for Rett syndrome
Source: Orphanet J Rare Dis. 2022 Mar 4;17:108. doi: 10.1186/s13023-022-02240-w (PMC8894842; doi:10.1186/s13023-022-02240-w)
Supplement: Supplementary file 1 — Additional file 1: Table S1. The use of outcome measures in clinical trials in Rett syndrome. [file 13023_2022_2240_MOESM1_ESM.docx]

**Supplementary Table 1. The use of outcome measures in clinical trials in Rett syndrome.**

| Measure | # of Trials | Trial Number/Trial Reference | Intervention | Trial Status | Validation data available for RTT | Phase of clinical trial |
| --- | --- | --- | --- | --- | --- | --- |
| Clinical Severity/Overall Development/Intellectual |  |  |  |  |  |  |
| Clinical Global Impression Scales (CGI) | 15 | NCT02715115  NCT01777542^1^  NCT01703533  NCT00261508^2^  NCT04304482  NCT04279314  NCT03941444  NCT04181723  NCT03758924  NCT03633058  NCT02696044  NCT02790034  NCT04252586  NCT03848832  Pini et al, 2012^3^ | NNZ-2566  rhIGF-1  NNZ-2566  Risperidone  ANAVEX2-73 (Blarcamesine)  Trofinetide  ANAVEX2-73 (Blarcamesine)  Trofinetide  ANAVEX2-73 (Blarcamesine)  Ketamine  Triheptanoin  Sarizotan  Cannabidiol (GWP42003-P)  Cannabidiol (GWP42003-P)  IGF-1 (Mecasermin-Increlex) | Complete  Complete  Complete  Complete  Recruiting  Recruiting  Recruiting  Recruiting  Complete  Recruiting  Recruiting  Terminated  Active (open label)  Terminated  Complete | No* | 2  2  2  3  2/3  3  2  3  2  2  2  2/3  3  3  1 |
| Rett Syndrome Motor and Behavioral Assessment (MBA) | 14 | NCT02715115  NCT01147575^4^  NCT01703533  NCT04041713  NCT04304482  NCT03633058  NCT02696044  NCT02790034  NCT04252586  NCT03848832  Hagebeuk et al, 2011^5^  Glaze et al, 2009^6^  Percy et al, 1994^7^  Chou et al, 2019^8^ | NNZ-2566  Creatine monohydrate  NNZ-2566  Rett-T (antioxidant combination)  ANAVEX2-73 (Blarcamesine)  Ketamine  Triheptanoin  Sarizotan  Cannabidiol (GWP42003-P)  Cannabidiol (GWP42003-P)  Folinic Acid  Folic Acid and Betaine  Naltrexone  Music Therapy | Complete  Complete  Complete  Not yet recruiting  Recruiting  Recruiting  Recruiting  Terminated  Recruiting  Terminated  Complete  Complete  Complete  Complete | Yes^9^ | 2  2  2  2  2/3  2  2  2/3  3  3  2  2  2  N/A |
| Rett Syndrome Clinical Severity Scale (CSS)^10^ | 7 | NCT01822249  NCT01703533  NCT02696044  NCT02790034  NCT03259971  De Felice et al, 2012^11^  Chou et al, 2019^8^ | EPI-743  NNZ-2566  Triheptanoin  Sarizotan  Probiotic (Lactobacillus planatarum)  ω-3 polyunsaturated fatty acids  Music Therapy | Complete  Complete  Recruiting  Terminated  Recruiting  Complete  Complete | No | 2  2  2  2  N/A  Pilot study  N/A |
| Mullen Scales of Early Learning (MSEL) | 5 | NCT01777542^1^  NCT01520363  NCT00593957^12^  NCT03259971  Martínez et al, 2013^13^ | rhIGF-1  Dextromethorphan  Dextromethorphan  Probiotic (Lactobacillus planatarum)  Venlafaxine/citalopram/risperidone | Complete  Complete  Terminated  Recruiting  Complete | Yes^$14^ | 2  2  2  N/A  N/A |
| Kerr Severity Scale/International Scoring Scale (ISS)^15^ | 4 | NCT02023424^16^  NCT01777542^1^  Pini et al, 2012^3^  Pini et al, 2016^17^ | Glatiramer acetate (Copaxone®)  rhIGF-1  IGF-1 (Mecasermin-Increlex)  rhIGF-1 (Mecasermin) | Terminated  Complete  Complete  Complete | No | 1  2  1  1 |
| Rett Assessment Rating Scale (RARS) | 3 | Fabio et al, 2018^18^  Gangemi et al, 2018^19^  Fabio et al, 2016^20^ | Long chain poly-unsaturated fatty acid  Transcranial direct current stimulation  Cognitive training | Complete  Complete  Complete | No | N/A  N/A  N/A |
| Modified Raven’s Coloured Progressive Matrices | 2 | Gangemi et al, 2018^19^  Fabio et al, 2016^20^ | Transcranial direct current stimulation  Cognitive training | Complete  Complete | No | N/A |
| Parent Global Impression Scales | 2 | NCT01777542^1^  NCT02696044 | rhIGF-1  Triheptanoin | Complete  Recruiting | No | 2  2 |
| Clinician Domain Likert Scale | 1 | NCT03633058 | Ketamine | Recruiting | No | 2 |
| Parent Domain Likert Scale | 1 | NCT03633058 | Ketamine | Recruiting | No | 2 |
| Caregiver-rated Impression of Change (CIC) | 1 | NCT02790034 | Sarizotan | Terminated | No | 2 |
| Rett Syndrome Severity Scale/Percy Scale^21^ | 1 | NCT02061137^22^ | Fingolimod | Complete | No | 1 |
| Rett Severity Score (RSS) | 1 | Pini et al, 2016^17^ | rhIGF-1 (Mecasermin) | Complete | No | 1 |
| Rett Severity Scale | 1 | NCT00593957^12^ | Dextromethorphan | Complete | No | 2 |
| Rett Syndrome: Symptom Severity Index (RS: SSI) | 1 | Ellaway et al, 2001^23^ | L-carnitine | Complete (open label) | No | N/A |
| Rett Functional Evaluation Scale | 1 | Lotan et al, 2012^24^ | Conductive environment | Complete | No^#^ | N/A |
| Bayley Scales of Infant Developmental Mental and Psychomotor Tests | 1 | Percy et al, 1994^7^ | Naltrexone | Complete | No | 2 |
| Motor (Gross and Fine) |  |  |  |  |  |  |
| Rett Syndrome Gross Motor Scale | 5 | NCT03848442^25^  NCT02696044  Downs et al, 2018^26^  Downs et al, 2012^27^  Lotan et al, 2012^24^ | ‘Uptime’ participation  Triheptanoin  Environmental enrichment  StepWatch Activity Monitor  Conductive environment | Complete  Recruiting  Complete  Complete  Complete | Yes^28^ | N/A  2  N/A  N/A  N/A |
| Hand Apraxia Scale | 4 | NCT02696044  NCT02061137^22^  Hagebeuk et al, 2011^5^  Ellaway et al, 2001^23^ | Triheptanoin  Fingolimod  Folinic Acid  L-carnitine | Recruiting  Complete  Complete  Complete (open label) | No^#^ | 2  1  2  N/A |
| Rett Syndrome Clinician Rating of Hand Function (RTT-HF) | 2 | NCT04279314  NCT04181723 | Trofinetide  Trofinetide | Recruiting  Recruiting | No | 3  3 |
| Rett Syndrome Clinician Rating of Ambulation and Gross Motor Skills (RTT-AMB) | 2 | NCT04279314  NCT04181723 | Trofinetide  Trofinetide | Recruiting  Recruiting | No | 3  3 |
| Burke-Fahn-Marsden Dystonia Rating Scale (BFM) | 2 | NCT02696044  NCT03259971 | Triheptanoin  Probiotic (Lactobacillus planatarum) | Recruiting  Recruiting | No | 2  N/A |
| Peabody Developmental Motor Scales (PDMS-2)/Peabody Fine Motor Developmental Schedules | 1 | Percy et al, 1994^7^ | Naltrexone | Complete | No | 2 |
| Global Dystonia Rating Scale (GDRS) | 1 | NCT02696044 | Triheptanoin | Recruiting | No | 2 |
| PROMIS Physical Functional Mobility Parent Proxy Score | 1 | NCT02696044 | Triheptanoin | Recruiting | No | 2 |
| PROMIS Physical Function Upper Extremity Parent Proxy Score | 1 | NCT02696044 | Triheptanoin | Recruiting | No | 2 |
| Hand Apraxia Scale Adapted for RTT | 1 | Lotan et al, 2012^24^ | Conductive environment | Complete | Yes^29^ | N/A |
| Gessell Gross and Fine Motor Assessment Scales | 1 | Percy et al, 1994^7^ | Naltrexone | Complete | No | 2 |
| Language |  |  |  |  |  |  |
| Communication and Symbolic Behavior Scales-Developmental Profile (CSBS-DP) | 3 | NCT01777542^1^  NCT04279314  NCT04181723 | rhIGF-1  Trofinetide  Trofinetide | Complete  Recruiting  Recruiting | No | 2  3  3 |
| Rett Syndrome Clinician Rating of Ability to Communicate Choices (RTT-COMC) | 2 | NCT04279314  NCT04181723 | Trofinetide  Trofinetide | Recruiting  Recruiting | No | 3  3 |
| Rett Syndrome Clinician Rating of Verbal Communication (RTT-VCOM) | 2 | NCT04279314  NCT04181723 | Trofinetide  Trofinetide | Recruiting  Recruiting | No | 3  3 |
| Fanzago Phonetic Articulation Test | 1 | Gangemi et al, 2018^19^ | Transcranial direct current stimulation | Complete | No | N/A |
| Sleep/Respiratory |  |  |  |  |  |  |
| Children’s Sleep Habits Questionnaire (CSHQ) | 7 | NCT04041713  NCT04304482  NCT03941444  NCT03758924  NCT03633058  NCT04252586  NCT03848832 | Rett-T (antioxidant combination)  ANAVEX2-73 (Blarcamesine)  ANAVEX2-73 (Blarcamesine)  ANAVEX2-73 (Blarcamesine)  Ketamine  Cannabidiol (GWP42003-P)  Cannabidiol (GWP42003-P) | Not yet recruiting  Recruiting  Recruiting  Complete  Recruiting  Recruiting  Active | No^ | 2  2/3  2  2  2  3  3 |
| Apnea-Hypopnea Index | 4 | NCT01777542^1^  NCT02153723^30^  NCT00990691^31^  NCT01253317^32^ | rhIGF-1  Glatiramer acetate (Copaxone®)  Desipramine  rhIGF-1 (Mecasermin) | Complete  Complete  Complete  Complete | No | 2  2  2  1 |
| Sleep Disturbance Scale for Children | 2 | NCT04167059  Downs et al, 2018^26^ | Telehealth participation strategies  Environmental enrichment | Recruiting  Complete | No | N/A  N/A |
| PROMIS Fatigue Parent Proxy Score | 1 | NCT02696044 | Triheptanoin | Recruiting | No | 2 |
| Respiratory Disturbance Index | 1 | NCT01822249 | EPI-743 | Complete | No | 2 |
| Social-Emotional-Behavioural |  |  |  |  |  |  |
| Rett Syndrome Behavior Questionnaire (RSBQ) | 16 | NCT01822249  NCT01777542^1^  NCT01520363  NCT04041713  NCT04304482  NCT04167059  NCT04279314  NCT03941444  NCT04181723  NCT03758924  NCT03633058  NCT02562820  NCT02696044  NCT04252586  NCT03848832  Downs et al, 2018^26^ | EPI-743  rhIGF-1  Dextromethorphan  Rett-T (antioxidant combination)  ANAVEX2-73 (Blarcamesine)  Telehealth participation strategies  Trofinetide  ANAVEX2-73 (Blarcamesine)  Trofinetide  ANAVEX2-73 (Blarcamesine)  Ketamine  Ketamine  Triheptanoin  Cannabidiol (GWP42003-P)  Cannabidiol (GWP42003-P)  Environmental enrichment | Complete  Complete  Complete  Not yet recruiting  Recruiting  Recruiting  Recruiting  Recruiting  Recruiting  Complete  Recruiting  Terminated  Recruiting  Recruiting  Active  Complete | Yes^33-36^ | 2  2  2  2  2/3  N/A  3  2  3  2  2  1  2  3  3  N/A |
| Vineland Adaptive Behavior Scales (VABS/VABS-2) | 11 | NCT01703533  NCT01520363  NCT03259971  NCT01777542^1^  NCT02061137^22^  Martínez et al, 2013^13^  Percy et al, 1994^7^  Fabio et al, 2018^18^  Chou et al, 2019^8^  Gangemi et al, 2018^19^  Fabio et al, 2016^20^ | NNZ-2566  Dextromethorphan  Probiotic (Lactobacillusplanatarum)  rhIGF-1  Fingolimod  Venlafaxine/citalopram/risperidone  Naltrexone  Long chain poly-unsaturated fatty acid  Music Therapy  Transcranial direct current stimulation  Cognitive training | Complete  Complete  Recruiting  Complete  Complete  Complete  Complete  Complete  Complete  Complete  Complete | No^!36^ | 2  2  N/A  2  1  N/A  2  N/A  N/A  N/A  N/A |
| Anxiety, Depression, and Mood Scale (ADAMS) | 7 | NCT01777542^1^  NCT01253317^32^  NCT04041713  NCT04304482  NCT03941444  NCT03758924  NCT03259971 | rhIGF-1  rhIGF-1 (Mecasermin)  Rett-T (antioxidant combination)  ANAVEX2-73 (Blarcamesine)  ANAVEX2-73 (Blarcamesine)  ANAVEX2-73 (Blarcamesine)  Probiotic (Lactobacillus planatarum) | Complete  Complete  Not yet recruiting  Recruiting  Recruiting  Complete  Recruiting | Yes^36^ | 2  1  2  2/3  2  2  N/A |
| Aberrant Behavior Checklist (ABC-C) | 5 | NCT01777542^1^  NCT01703533  NCT00261508^2^  NCT00593957^12^  Martínez et al, 2013^13^ | rhIGF-1  NNZ-2566  Risperidone  Dextromethorphan  Venlafaxine/citalopram/risperidone | Complete  Complete  Complete  Complete  Complete | Yes^36^ | 2  2  3  2  N/A |
| Ghuman-Folstein Screen for Social Interaction (SSI) | 3 | NCT01520363  NCT00593957^12^  NCT03259971 | Dextromethorphan  Dextromethorphan  Probiotic (Lactobacillus planatarum) | Complete  Complete  Recruiting | No | 2  2  N/A |
| Nisonger Child Behavior Rating Form (N-CBRF) | 1 | NCT00261508^2^ | Risperidone | Complete | No | 3 |
| PROMIS Peer Relations Parent Proxy Score | 1 | NCT02696044 | Triheptanoin | Recruiting | No | 2 |
| PROMIS Anxiety Parent Proxy Score | 1 | NCT02696044 | Triheptanoin | Recruiting | No | 2 |
| PROMIS Depressive Symptoms Parent Proxy Score | 1 | NCT02696044 | Triheptanoin | Recruiting | No | 2 |
| Early Social Communication Scales | 1 | NCT03259971 | Probiotic (Lactobacillus planatarum) | Recruiting | No | N/A |
| Quality of Life/Health/Disability |  |  |  |  |  |  |
| PedsQL | 3 | NCT01822249  NCT01520363  NCT02428673 | EPI-743  Dextromethorphan  Assisted Standing Treatment Program | Complete  Complete  Complete | No^!37^ | 2  2  N/A |
| Child Health Questionnaire (CHQ) | 2 | NCT02153723^30^  NCT02696044 | Glatiramer acetate (Copaxone®)  Triheptanoin | Complete  Recruiting | Yes^38^ | 2  2 |
| Quality of Life Inventory-Disability | 2 | NCT03848442^25^  NCT04167059 | ‘Uptime’ participation  Telehealth participation strategies | Complete  Recruiting | Yes^39-41^ | N/A  N/A |
| Impact of Childhood Neurologic Disability Scale (ICND) | 2 | NCT04279314  NCT04181723 | Trofinetide  Trofinetide | Recruiting  Recruiting | No | 3  3 |
| Pediatric Evaluation of Disability Inventory (PEDI) | 1 | NCT03259971 | Probiotic (Lactobacillus planatarum) | Recruiting | No | N/A |
| 36-Item Short Form Survey (SF-36) | 1 | Ellaway et al, 2001^23^ | L-carnitine | Complete (open label) | No | N/A |
| Caregiver Priorities and Child Health Index of Life with Disabilities (CPCHILD) | 1 | NCT02428673 | Assisted Standing Treatment Program | Active | No | N/A |
| PROMIS Pain Interference Parent Proxy Score | 1 | NCT02696044 | Triheptanoin | Recruiting | No | 2 |
| Parental/Caregiver Concern |  |  |  |  |  |  |
| Caregiver/Parent Targeted Visual Analog Scale/Visual Analog Scale (PTSVAS/VAS) | 8 | NCT02715115  NCT01777542^1^  NCT01703533  NCT00261508^2^  NCT04041713  NCT03758924  NCT02696044  NCT02790034 | NNZ-2566  rhIGF-1  NNZ-2566  Risperidone  Rett-T (antioxidant combination)  ANAVEX2-73 (Blarcamesine)  Triheptanoin  Sarizotan | Complete  Complete  Complete  Complete  Not yet recruiting  Complete  Recruiting  Terminated | No | 2  2  2  3  2  2  2  2/3 |
| Rett Syndrome Caregiver Burden Inventory Assessment (RTT-CBI) | 3 | NCT04279314  NCT04181723  NCT03633058 | Trofinetide  Trofinetide  Ketamine | Recruiting  Recruiting  Recruiting | Yes^42^ | 3  3  2 |
| Goal Attainment Scaling | 1 | NCT03848442^25^ | ‘Uptime’ participation | Complete | No | N/A |
| Parental Overall Well-being Index | 1 | Hagebeuk et al, 2011^5^ | Folinic Acid | Complete | No | 2 |
| Parenting Stress Index | 1 | Chou et al, 2019^8^ | Music Therapy | Complete | No | N/A |

*Rett-specific anchors have been developed for CGI but it has not undergone rigorous validation studies^43^

$ Adapted MSEL has been validated for RTT

^CSHQ has shown strong internal consistency in a small sample in RTT^44^

# Rett Functional Evaluation Scale has been shown to have inter-rater reliability in a study of four girls with RTT^45^

^!^ Validated in children and adults with intellectual disability^36,39-41^
