# Supplementary material for: Improving clinical trial readiness to accelerate development of new therapeutics for Rett syndrome
Source: Orphanet J Rare Dis. 2022 Mar 4;17:108. doi: 10.1186/s13023-022-02240-w (PMC8894842; doi:10.1186/s13023-022-02240-w)
Supplement: Supplementary file 2 — Additional file 2: References for Table S1. [file 13023_2022_2240_MOESM2_ESM.docx]

**References**

1. O'Leary HM, Kaufmann WE, Barnes KV, Rakesh K, Kapur K, Tarquinio DC, et al. Placebo-controlled crossover assessment of mecasermin for the treatment of Rett syndrome. Ann Clin Transl Neurol. 2018 Mar;5(3):323-332. doi: 10.1002/acn3.533

2. Shea S, Turgay A, Carroll A, Schulz M, Orlik H, Smith I, et al. Risperidone in the treatment of disruptive behavioral symptoms in children with autistic and other pervasive developmental disorders. Pediatrics. 2004 Nov;114(5):e634-41. doi: 10.1542/peds.2003-0264-F

3. Pini G, Scusa MF, Congiu L, Benincasa A, Morescalchi P, Bottiglioni I, et al. IGF1 as a Potential Treatment for Rett Syndrome: Safety Assessment in Six Rett Patients. Autism Res Treat. 2012;2012:679801. doi: 10.1155/2012/679801

4. Freilinger M, Dunkler D, Lanator I, Item CB, Mühl A, Fowler B, et al. Effects of creatine supplementation in Rett syndrome: a randomized, placebo-controlled trial. J Dev Behav Pediatr. 2011 Jul-Aug;32(6):454-60. doi: 10.1097/DBP.0b013e31822177a8

5. Hagebeuk EE, Duran M, Koelman JH, Abeling NG, Vyth A, Poll-The BT. Folinic acid supplementation in Rett syndrome patients does not influence the course of the disease: a randomized study. J Child Neurol. 2012 Mar;27(3):304-9. doi: 10.1177/0883073811417184

6. Glaze DG, Percy AK, Motil KJ, Lane JB, Isaacs JS, Schultz RJ, et al. A study of the treatment of Rett syndrome with folate and betaine. J Child Neurol. 2009 May;24(5):551-6. doi: 10.1177/0883073808327827

7. Percy AK, Glaze DG, Schultz RJ, Zoghbi HY, Williamson D, Frost JD, Jr., et al. Rett syndrome: controlled study of an oral opiate antagonist, naltrexone. Ann Neurol. 1994 Apr;35(4):464-70. doi: 10.1002/ana.410350415

8. Chou MY, Chang NW, Chen C, Lee WT, Hsin YJ, Siu KK, et al. The effectiveness of music therapy for individuals with Rett syndrome and their families. J Formos Med Assoc. 2019 Dec;118(12):1633-1643. doi: 10.1016/j.jfma.2019.01.001

9. Raspa M, Bann CM, Gwaltney A, Benke TA, Fu C, Glaze DG, et al. A Psychometric Evaluation of the Motor-Behavioral Assessment Scale for Use as an Outcome Measure in Rett Syndrome Clinical Trials. Am J Intellect Dev Disabil. 2020 Nov 1;125(6):493-509. doi: 10.1352/1944-7558-125.6.493

10. Neul JL, Fang P, Barrish J, Lane J, Caeg EB, Smith EO, et al. Specific mutations in methyl-CpG-binding protein 2 confer different severity in Rett syndrome. Neurology. 2008 Apr 15;70(16):1313-21. doi: 10.1212/01.wnl.0000291011.54508.aa

11. De Felice C, Signorini C, Durand T, Ciccoli L, Leoncini S, D'Esposito M, et al. Partial rescue of Rett syndrome by ω-3 polyunsaturated fatty acids (PUFAs) oil. Genes Nutr. 2012 Jul;7(3):447-58. doi: 10.1007/s12263-012-0285-7

12. Smith-Hicks CL, Gupta S, Ewen JB, Hong M, Kratz L, Kelley R, et al. Randomized open-label trial of dextromethorphan in Rett syndrome. Neurology. 2017 Oct 17;89(16):1684-1690. doi: 10.1212/wnl.0000000000004515

13. Martínez A, Turon M, Callejón-Póo L, Sole E, Armstrong J, Pineda M. Treatment response in behaviour disorders in rett syndrome. J Behav Brain Sci. 2013;3(2):217-224. doi: <http://dx.doi.org/10.4236/jbbs.2013.32023>

14. Clarkson T, LeBlanc J, DeGregorio G, Vogel-Farley V, Barnes K, Kaufmann WE, et al. Adapting the Mullen Scales of Early Learning for a Standardized Measure of Development in Children With Rett Syndrome. Intellect Dev Disabil. 2017 Dec;55(6):419-431. doi: 10.1352/1934-9556-55.6.419

15. Kerr AM, Nomura Y, Armstrong D, Anvret M, Belichenko PV, Budden S, et al. Guidelines for reporting clinical features in cases with MECP2 mutations. Brain Dev. 2001 Jul;23(4):208-11. doi: 10.1016/s0387-7604(01)00193-0

16. Nissenkorn A, Kidon M, Ben-Zeev B. A Potential Life-Threatening Reaction to Glatiramer Acetate in Rett Syndrome. Pediatr Neurol. 2017 Mar;68:40-43. doi: 10.1016/j.pediatrneurol.2016.11.006

17. Pini G, Congiu L, Benincasa A, DiMarco P, Bigoni S, Dyer AH, et al. Illness Severity, Social and Cognitive Ability, and EEG Analysis of Ten Patients with Rett Syndrome Treated with Mecasermin (Recombinant Human IGF-1). Autism Research and Treatment. 2016 2016/01/26;2016:5073078. Available from: <https://doi.org/10.1155/2016/5073078> doi: 10.1155/2016/5073078

18. Fabio R, Martino G, Caprì T, Giacchero R, Giannatiempo S, Antonietti A, et al. Long chain poly-unsaturated fatty acid supplementation in Rett syndrome: a randomized placebo-controlled trial. Asian J Clin Nutr. 2018;10:37-46.

19. Gangemi A, Caprí T, Fabio R, Puggioni P, Falzone A, Martino G. Transcranial direct current stimulation (TDCS) and cognitive empowerment for the functional recovery of diseases with chronic impairment and genetic etiopathogenesis. . In: Urbano K, editor. Advances in Genetics Research. New York, USA: Nova Science Publishers; 2018.

20. Fabio RA, Billeci L, Crifaci G, Troise E, Tortorella G, Pioggia G. Cognitive training modifies frequency EEG bands and neuropsychological measures in Rett syndrome. Res Dev Disabil. 2016 Jun-Jul;53-54:73-85. doi: 10.1016/j.ridd.2016.01.009

21. Schanen C, Houwink EJ, Dorrani N, Lane J, Everett R, Feng A, et al. Phenotypic manifestations of MECP2 mutations in classical and atypical Rett syndrome. Am J Med Genet A. 2004 Apr 15;126a(2):129-40. doi: 10.1002/ajmg.a.20571

22. Naegelin Y, Kuhle J, Schädelin S, Datta AN, Magon S, Amann M, et al. Fingolimod in children with Rett syndrome: the FINGORETT study. Orphanet J Rare Dis. 2021 Jan 6;16(1):19. doi: 10.1186/s13023-020-01655-7

23. Ellaway CJ, Peat J, Williams K, Leonard H, Christodoulou J. Medium-term open label trial of L-carnitine in Rett syndrome. Brain Dev. 2001 Dec;23 Suppl 1:S85-9. doi: 10.1016/s0387-7604(01)00346-1

24. Lotan M, Schenker R, Wine J, Downs J. The conductive environment enhances gross motor function of girls with Rett syndrome. A pilot study. Dev Neurorehabil. 2012;15(1):19-25. doi: 10.3109/17518423.2011.629374

25. Stahlhut M, Downs J, Wong K, Bisgaard AM, Nordmark E. Feasibility and Effectiveness of an Individualized 12-Week "Uptime" Participation (U-PART) Intervention in Girls and Women With Rett Syndrome. Phys Ther. 2020 Jan 23;100(1):168-179. doi: 10.1093/ptj/pzz138

26. Downs J, Rodger J, Li C, Tan X, Hu N, Wong K, et al. Environmental enrichment intervention for Rett syndrome: an individually randomised stepped wedge trial. Orphanet J Rare Dis. 2018 Jan 10;13(1):3. doi: 10.1186/s13023-017-0752-8

27. Downs J, Leonard H, Hill K. Initial assessment of the StepWatch Activity Monitor™ to measure walking activity in Rett syndrome. Disabil Rehabil. 2012;34(12):1010-5. doi: 10.3109/09638288.2011.630773

28. Downs J, Stahlhut M, Wong K, Syhler B, Bisgaard AM, Jacoby P, et al. Validating the Rett Syndrome Gross Motor Scale. PLoS One. 2016;11(1):e0147555. doi: 10.1371/journal.pone.0147555

29. Downs J, Bebbington A, Jacoby P, Williams AM, Ghosh S, Kaufmann WE, et al. Level of purposeful hand function as a marker of clinical severity in Rett syndrome. Dev Med Child Neurol. 2010 Sep;52(9):817-23. doi: 10.1111/j.1469-8749.2010.03636.x

30. Djukic A, Holtzer R, Shinnar S, Muzumdar H, Rose SA, Mowrey W, et al. Pharmacologic Treatment of Rett Syndrome With Glatiramer Acetate. Pediatr Neurol. 2016 Aug;61:51-7. doi: 10.1016/j.pediatrneurol.2016.05.010

31. Mancini J, Dubus JC, Jouve E, Roux JC, Franco P, Lagrue E, et al. Effect of desipramine on patients with breathing disorders in RETT syndrome. Ann Clin Transl Neurol. 2018 Feb;5(2):118-127. doi: 10.1002/acn3.468

32. Khwaja OS, Ho E, Barnes KV, O'Leary HM, Pereira LM, Finkelstein Y, et al. Safety, pharmacokinetics, and preliminary assessment of efficacy of mecasermin (recombinant human IGF-1) for the treatment of Rett syndrome. Proc Natl Acad Sci U S A. 2014 Mar 25;111(12):4596-601. doi: 10.1073/pnas.1311141111

33. Hou W, Bhattacharya U, Pradana WA, Tarquinio DC. Assessment of a Clinical Trial Metric for Rett Syndrome: Critical Analysis of the Rett Syndrome Behavioural Questionnaire. Pediatr Neurol. 2020 Jun;107:48-56. doi: 10.1016/j.pediatrneurol.2020.01.009

34. Oberman LM, Downs J, Cianfaglione R, Leonard H, Kaufmann WE. Assessment of a Clinical Trial Metric for Rett Syndrome: Critical Analysis of the Rett Syndrome Behaviour Questionnaire. Pediatr Neurol. 2020 Oct;111:4. doi: 10.1016/j.pediatrneurol.2020.04.020

35. Mount RH, Charman T, Hastings RP, Reilly S, Cass H. The Rett Syndrome Behaviour Questionnaire (RSBQ): refining the behavioural phenotype of Rett syndrome. J Child Psychol Psychiatry. 2002 Nov;43(8):1099-110. doi: 10.1111/1469-7610.00236

36. Barnes KV, Coughlin FR, O'Leary HM, Bruck N, Bazin GA, Beinecke EB, et al. Anxiety-like behavior in Rett syndrome: characteristics and assessment by anxiety scales. J Neurodev Disord. 2015;7(1):30. doi: 10.1186/s11689-015-9127-4

37. Viecili MA, Weiss JA. Reliability and Validity of the Pediatric Quality of Life Inventory With Individuals With Intellectual and Developmental Disabilities. Am J Intellect Dev Disabil. 2015 Jul;120(4):289-301. doi: 10.1352/1944-7558-120.4.289

38. Lane JB, Lee HS, Smith LW, Cheng P, Percy AK, Glaze DG, et al. Clinical severity and quality of life in children and adolescents with Rett syndrome. Neurology. 2011 Nov 15;77(20):1812-8. doi: 10.1212/WNL.0b013e3182377dd2

39. Jacoby P, Epstein A, Kim R, Murphy N, Leonard H, Williams K, et al. Reliability of the Quality of Life Inventory-Disability Measure in Children with Intellectual Disability. J Dev Behav Pediatr. 2020 Sep;41(7):534-539. doi: 10.1097/dbp.0000000000000815

40. Epstein A, Williams K, Reddihough D, Murphy N, Leonard H, Whitehouse A, et al. Content validation of the Quality of Life Inventory-Disability. Child Care Health Dev. 2019 Sep;45(5):654-659. doi: 10.1111/cch.12691

41. Downs J, Jacoby P, Leonard H, Epstein A, Murphy N, Davis E, et al. Psychometric properties of the Quality of Life Inventory-Disability (QI-Disability) measure. Qual Life Res. 2019 Mar;28(3):783-794. doi: 10.1007/s11136-018-2057-3

42. Lane JB, Salter AR, Jones NE, Cutter G, Horrigan J, Skinner SA, et al. Assessment of Caregiver Inventory for Rett Syndrome. J Autism Dev Disord. 2017 Apr;47(4):1102-1112. doi: 10.1007/s10803-017-3034-3

43. Neul JL, Glaze DG, Percy AK, Feyma T, Beisang A, Dinh T, et al. Improving Treatment Trial Outcomes for Rett Syndrome: The Development of Rett-specific Anchors for the Clinical Global Impression Scale. J Child Neurol. 2015 Nov;30(13):1743-8. doi: 10.1177/0883073815579707

44. Merbler AM, Byiers BJ, Garcia JJ, Feyma TJ, Symons FJ. The feasibility of using actigraphy to characterize sleep in Rett syndrome. J Neurodev Disord. 2018 Feb 27;10(1):8. doi: 10.1186/s11689-018-9227-z

45. Lotan M, Isakov E, Merrick J. Improving functional skills and physical fitness in children with Rett syndrome. J Intellect Disabil Res. 2004 Nov;48(Pt 8):730-5. doi: 10.1111/j.1365-2788.2003.00589.x
